# Traumatic combined vertical atlanto-occipital and atlanto-axial dislocations with 2-part fracture of the atlas: Two case reports

**DOI:** 10.1097/MD.0000000000017776

**Published:** 2019-11-01

**Authors:** Jong-Beom Park, Dong-Gune Chang, Whoan Jeang Kim, Eung Sic Kim

**Affiliations:** a Department of Orthopaedic Surgery, Uijeongbu St. Mary's Hospital, The Catholic University of Korea, Gyeonggi-do; b Department of Orthopaedic Surgery, Sanggye Paik Hospital, College of Medicine, Inje University, Seoul; c Department of Orthopaedic Surgery, Eulji University Hospital, Daejeon, Korea.

**Keywords:** 2-part fracture of the atlas, vertical atlanto-axial dislocation, Vertical atlanto-occipital dislocation

## Abstract

**Rationale:**

To our knowledge, this is the first report of traumatic combined vertical atlanto-occipital dislocation (AOD) and atlanto-axial dislocation (AAD) with 2-part fracture of the atlas.

**Patient concerns:**

The first case was of a 31-year-old woman admitted to the emergency room comatose after a traffic accident. The second case was of a 21-year-old woman admitted to the emergency room comatose after a fall.

**Diagnoses:**

Traumatic combined vertical AOD and AAD with 2-part fractures of the atlas was diagnosed using plain radiography, 2-dimensional computed tomography, and/or magnetic resonance imaging of the cervical spine.

**Intervention:**

The first patient received immediate intubation and cardiopulmonary resuscitation in the emergency room. The second patient also received immediate intubation in the emergency room. After her vitals stabilized, she underwent occipitocervical fusion with instrumentation.

**Outcomes:**

The first patient died 2 days after the accident. The second patient remained quadriplegic in a ventilatory-dependent state at 1 year after surgery. She continues to receive comprehensive rehabilitation.

**Lessons:**

Immediate respiratory support and surgical stabilization are important for saving lives in this kind of extremely unstable and fatal complex upper cervical spine injury.

## 1 Introduction

Traumatic atlanto-occipital dislocation (AOD) is a rare but usually life-threatening injury. Immediate diagnosis and surgical stabilization are essential for preventing further neurologic deterioration or death.^[1,2]^ To date, 4 types of AOD have been reported: anterior, posterior, lateral, and vertical (or distractive). Among them, vertical-type AOD is seemingly the most unstable and fatal because of a complete rupture of ligamentous structures of the occipito-cervical junction. The mechanism of injury in vertical AOD is thought to be a distraction and/or hyperextension force.^[3–5]^ Notably, the spinal cord is more vulnerable to distraction forces than compression forces.^[1,2]^ Such distraction and/or hyperextension forces can also cause vertical AOD and horizontal split fracture of the anterior arch of the atlas.^[1–5]^ Survivors of vertical-type AOD are rare compared with those of the other 3 types of AOD.^[3–5]^

AOD can occur either alone or in conjunction with other cervical spine fractures or dislocations.^[6,7]^ Few studies to date have reported combined injury of atlanto-occipital and atlanto-axial joints.^[8–11]^ According to our literature review, traumatic combined vertical AOD and atlanto-axial dislocation (AAD) with 2-part fracture of the atlas including horizontal split fracture of the anterior arch has not yet been reported. Therefore, the diagnosis, treatment, and prognosis of this type of extremely rare complex upper cervical spine injury remain poorly defined.

## 2 Case report

### 2.1 Case 1

**Figure 1:**
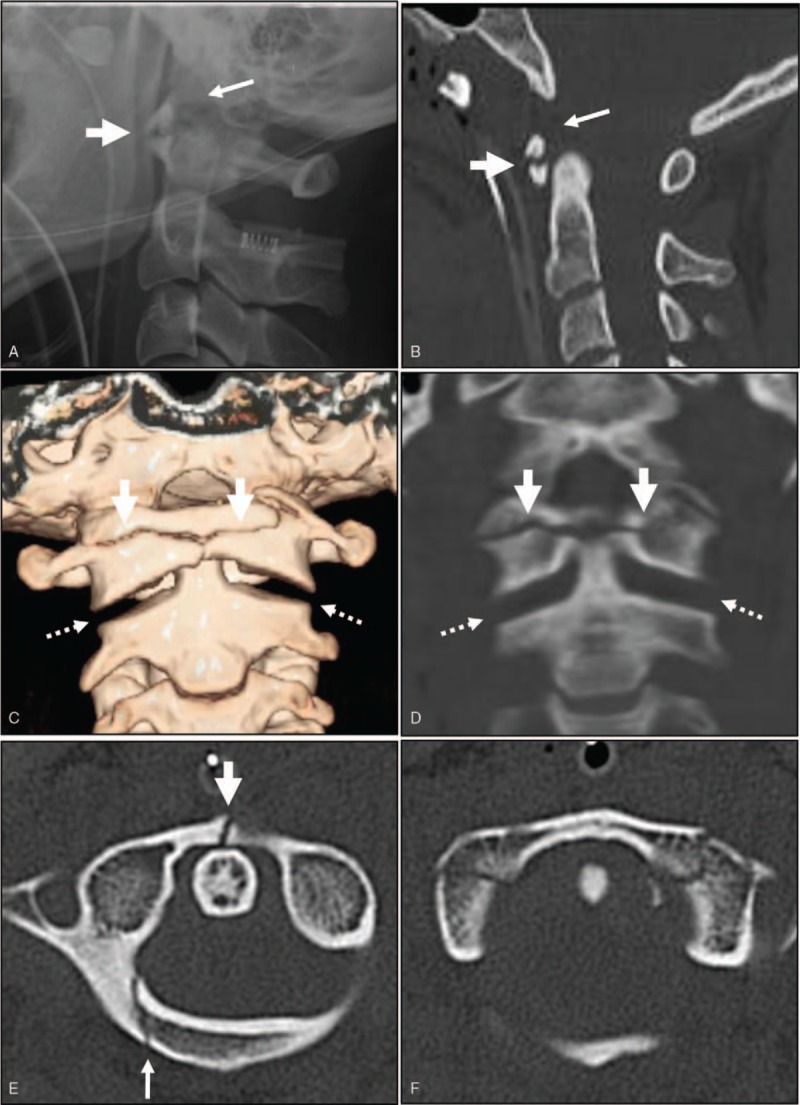
Lateral radiograph and sagittal 2-D computed tomography (CT) revealed horizontal split fracture and upward displacement of the anterior arch of the atlas (thick arrow), as well as an increased basion-dental interval of 18 mm (arrow) (A and B). Coronal 3-D and 2-D CT revealed horizontal split fracture of the anterior arch of the atlas (thick arrow) and increased atlanto-axial joints (dotted arrows) (C and D). Axial CT revealed 2-part fracture of the atlas including anterior arch fracture (thick arrow) and posterior arch fracture (arrow) (E and F).

A 31-year-old woman was admitted to the emergency room comatose following a traffic accident. Her cervical spine was immobilized in a Philadelphia cervical collar brace in the neutral position at the scene. She was unresponsive and had a Glasgow Coma Score (GCS) of 3. The polytrauma was scored as Injury Severity Score (ISS) 41. It was impossible to determine the status of her motor and sensory functions. She received immediate endotracheal intubation and insertion of a chest tube and L-tube. Aggressive fluid resuscitation and dopamine were required because of hypotension. Fluids administered included packed red blood cells and fresh frozen plasma. Following the connection of a ventilator in continuous mandatory ventilation (CMV) mode (tidal volume [TV] 480 and fraction of inspired oxygen [FiO_2_] 0.8), the patient was moved to the intensive care unit (ICU). Lateral radiograph and sagittal 2-dimensional (2-D) computed tomography (CT) revealed a horizontal split fracture and upward displacement of the anterior arch of the atlas as well as an increased basin-dental interval of 18 mm (Fig. 1A and B). Coronal 3-D and 2-D CT revealed horizontal and coronal split fractures of the anterior arch of the atlas and increased atlanto-axial joints (Fig. 1C and D**)**. Axial CT revealed a 2-part fracture of the atlas, including anterior and posterior arch fractures (Fig. 1E and F**)**. Furthermore, the patient had sustained a hemopeumothorax, spleen laceration, lungs and liver contusions, and hypoxic brain injury. Two days after the traffic accident, the patient was diagnosed with brain death. The patient's organs were subsequently donated to other patients after her death.

### 2.2 Case 2

A 21-year-old woman was admitted to the emergency room comatose after a fall. She was unresponsive, with a GCS of 3 and an ISS 38 polytrauma score. It was impossible to determine the status of her motor and sensory functions. She received immediate endotracheal intubation and insertion of a chest tube and L-tube. Aggressive fluid resuscitation and dopamine were required because of hypotension. Fluids administered included packed red blood cells and fresh frozen plasma. After the connection of ventilator in CMV mode (TV 480 and FiO_2_ 0.8), the patient was moved to the ICU. Lateral radiograph of the cervical spine revealed increased soft tissue swelling, a horizontal split fracture of the anterior arch of the atlas, and an increased bastion-dental interval of 24 mm (Fig. 2 A). Sagittal magnetic resonance imaging (MRI) revealed complete rupture of the ligamentous complex of the occipito-cervical junction and severe intramedullary cord hemorrhage at the occipito-cervical junction (Fig. 2B). Coronal 2-D CT revealed vertical displacement of the occipital condyles and increased atlanto-axial joints (Fig. 2 C). Sagittal 2-D CT revealed a horizontal split fracture of the anterior arch of the atlas, increased atlanto-axial joints, and an increased basion-dental interval of 24 mm (Fig. 2 D). Axial CT demonstrated a 2-part fracture of the atlas including anterior and posterior arch fractures (Fig. 2 E and F). Parasagittal 2-D CT revealed vertical displacement of the occipital condyles with respect to the lateral mass of the atlas and posterior arch fracture of the atlas (Fig. 2 G and H). Furthermore, the patient sustained a pneumothorax, liver contusion, and subarachnoid hemorrhage. When the patient's vitals stabilized, she underwent occipitocervical fusion using the Prima occipito-cervico-thoracic (OCT) system (U & I Corporation, Korea) and autogenous iliac bone graft at 26 days after the accident. At 1 year post-surgery, plain radiographs of the cervical spine revealed solid fusion of the occipitocervical junction with maintenance with the Prima OCT system (Fig. 2 I and J). However, the patient remains quadriplegic in a ventilatory-dependent state and continues to receive comprehensive rehabilitation.

**Figure 2:**
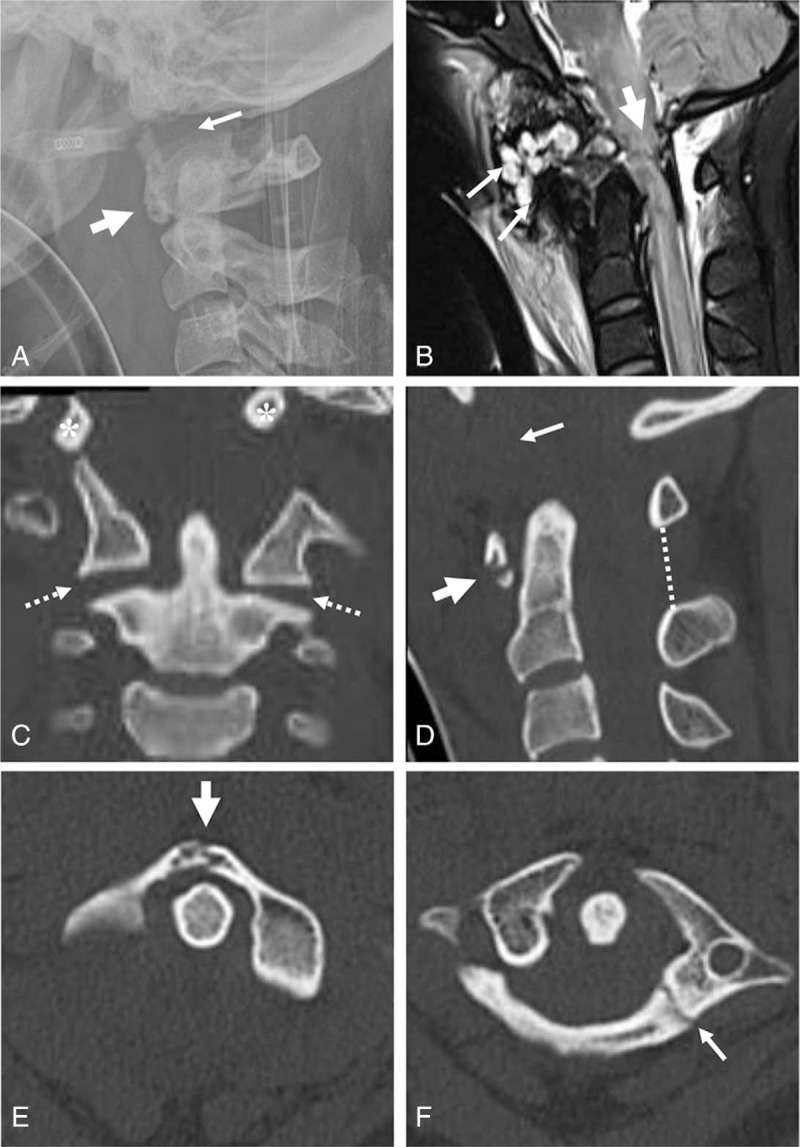
Lateral radiograph of the cervical spine revealed increased soft tissue swelling, horizontal split fracture of the anterior arch of the atlas (thick arrow), and increased basion-dental interval of 24 mm (arrow) (A). Sagittal MRI revealed complete rupture of the ligamentous complex (white arrows) and severe intramedullary cord hemorrhage at the occipito-cervical junction (thick arrow) (B). Coronal 2-D CT revealed vertical displacement of the occipital condyles (asterisks), and increased atlanto-axial joints (dotted arrows) (**C**). Sagittal 2-D CT revealed horizontal split fracture of the anterior arch of the atlas (thick arrows), increased atlanto-axial joints (dotted arrows), and increased basion-dental interval of 24 mm (arrow) (**D**). Axial CT revealed 2-part fracture of the atlas, including anterior arch fracture (thick arrow) and posterior arch fracture (arrow) (E and F). Parasagittal 2-D CT scans demonstrated vertical displacement of the occipital condyles (asterisks) with respect to the lateral mass of the atlas and posterior arch fracture of the atlas (arrowhead) (G and H). At 1 year post-surgery, plain radiographs of the cervical spine revealed solid fusion of the occipitocervical junction (I and J).

**Figure 2 (Continued):**
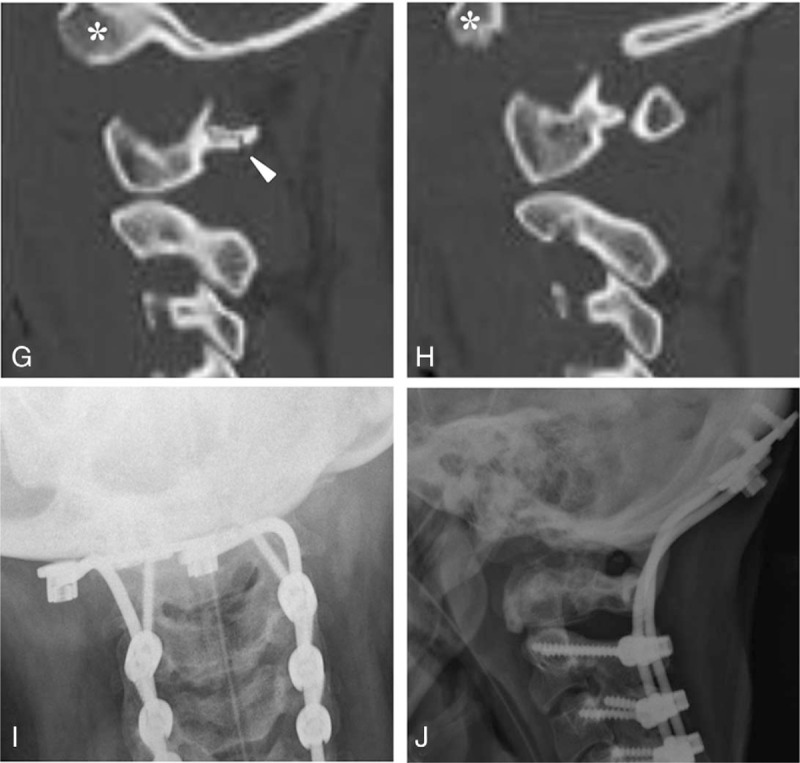
Lateral radiograph of the cervical spine revealed increased soft tissue swelling, horizontal split fracture of the anterior arch of the atlas (thick arrow), and increased basion-dental interval of 24 mm (arrow) (A). Sagittal MRI revealed complete rupture of the ligamentous complex (white arrows) and severe intramedullary cord hemorrhage at the occipito-cervical junction (thick arrow) (B). Coronal 2-D CT revealed vertical displacement of the occipital condyles (asterisks), and increased atlanto-axial joints (dotted arrows) (**C**). Sagittal 2-D CT revealed horizontal split fracture of the anterior arch of the atlas (thick arrows), increased atlanto-axial joints (dotted arrows), and increased basion-dental interval of 24 mm (arrow) (**D**). Axial CT revealed 2-part fracture of the atlas, including anterior arch fracture (thick arrow) and posterior arch fracture (arrow) (E and F). Parasagittal 2-D CT scans demonstrated vertical displacement of the occipital condyles (asterisks) with respect to the lateral mass of the atlas and posterior arch fracture of the atlas (arrowhead) (G and H). At 1 year post-surgery, plain radiographs of the cervical spine revealed solid fusion of the occipitocervical junction (I and J).

## 3 Discussion

AOD is a rare but usually life-threatening injury. To date, 4 types of AOD have been reported: anterior, posterior, lateral, and vertical (or distractive).^[1–5]^ Of these, vertical-type AOD is seemingly the most unstable and fatal because of complete rupture of the ligamentous structures of the occipitocervical junction. Of note, the spinal cord is more vulnerable to distraction forces than compression forces.^[2]^ As a result, there are fewer survivors of vertical-type AOD compared with the other three types of AOD.^[12]^ Moreover, the 2 cases of vertical-type AOD presented herein were accompanied by vertical AAD and 2-part fracture of the atlas, as well as injuries to the brain, chest, and abdomen. Accordingly, this complex upper cervical spine injury is thought to be the most unstable and fatal type compared with any other type of upper cervical spine injury.

Immediate diagnosis and surgical stabilization are essential for preventing further neurologic deterioration or death in AOD. Several radiologic parameters have been suggested to be useful in identifying AOD.^[13–16]^ The basion-dental interval is a reliable imaging method to detect AOD. A distance >10 mm is highly suggestive of AOD. This was shown in our 2 cases.^[17]^ Additionally, plain radiograph of AAD showed a posterior dislocation of the atlas with respect to the axis.^[18]^ However, plain radiographs can also easily overlook the diagnosis of AOD and AAD.^[6]^ Therefore, as we demonstrated in our 2 cases, a 2-D CT is the most powerful method for diagnosing of this kind of complex upper cervical spine injury. MRI is also helpful for diagnosing injuries of the spinal cord and ligamentous structures of the occipitocervical junction, as shown in the second case. In the present study, we found 2 common radiologic findings, which were accompanied by 2 cases of traumatic vertical AOD. Both cases had vertical AAD and horizontal split fractures of the anterior arch of the atlas. The mechanism of injury of vertical AOD is thought to be a distraction and/or hyperextension forces.^[15,16]^ Such distraction and/or hyperextension forces can also cause vertical AOD and horizontal split fracture of the anterior arch of the atlas. Because vertical AAD is a very rare injury, it can easily be overlooked if it is accompanied by vertical AOD. Therefore, these findings suggest that if we identify horizontal split fracture of the C1 anterior arch in a vertical-type AOD, we should pay more attention to the possibility of accompanying vertical AOD.

Occipitocervical fusion with instrumentation is the choice of surgery for the treatment of combined vertical AOD and AAD and 2-part fracture of the atlas.^[8–11]^ Preoperative skull traction is an absolute contraindication because it aggravates any vertical dislocation of the atlanto-occipital and atlanto-axial joints. Immediate intubation is necessary to prevent respiratory arrest because vertical AOD and/or AAD can cause spinal cord injury at the occipito-cervical junction above C3. However, the prognosis of combined vertical AOD and AAD seems unfavorable. In our 2 cases, 1 patient died at 2 days after her accident and the other remained quadriplegic and in a ventilatory-dependent state at 1 year post-surgery. Therefore, it is very important to provide enough explanation to patients and their families and to obtain informed consent before treatment and surgery, particularly in cases of poor prognosis.

In conclusion, this is believed to be the first study to report traumatic combined vertical AOD and AAD with 2-part fracture of the atlas including horizontal split fracture of the anterior arch. Immediate respiratory support and surgical stabilization are essential for saving lives in this kind of extremely unstable and fatal complex upper cervical spine injury. Horizontal split fracture of the anterior arch of the atlas can be a diagnostic clue for traumatic combined vertical AOD and AAD.

## Author contributions

**Conceptualization:** Jong-Beom Park, Dong-Gune Chang.

**Data curation:** Jong-Beom Park, Whoan Jeang Kim.

**Investigation:** Eung Sic Kim.

**Methodology:** Whoan Jeang Kim.

**Project administration:** Dong-Gune Chang.

**Resources:** Jong-Beom Park.

**Supervision:** Jong-Beom Park, Dong-Gune Chang.

**Validation:** Dong-Gune Chang, Eung Sic Kim.

**Visualization:** Dong-Gune Chang.

**Writing – original draft:** Jong-Beom Park.

**Writing – review & editing:** Dong-Gune Chang.
